# Association between cytokine response, the LRINEC score and outcome in patients with necrotising soft tissue infection: a multicentre, prospective study

**DOI:** 10.1038/srep42179

**Published:** 2017-02-08

**Authors:** Marco Bo Hansen, Lars Simon Rasmussen, Mattias Svensson, Bhavya Chakrakodi, Trond Bruun, Martin Bruun Madsen, Anders Perner, Peter Garred, Ole Hyldegaard, Anna Norrby-Teglund, INFECT study group, Michael Nekludov, Per Arnell, Anders Rosén, Nicklas Oscarsson, Ylva Karlsson, Oddvar Oppegaard, Steinar Skrede, Andreas Itzek, Anna Mygind Wahl, Morten Hedetoft, Nina Falcon Bærnthsen, Rasmus Müller, Torbjørn Nedrebø

**Affiliations:** 1 Department of Anaesthesia, Centre of Head and Orthopedics, Rigshospitalet, University of Copenhagen, Copenhagen, Denmark; 2 Hyperbaric Unit, Department of Anaesthesia, Centre of Head and Orthopedics, Rigshospitalet, University of Copenhagen, Copenhagen, Denmark; 3 Center for Infectious Medicine, Karolinska Institutet, Karolinska University Hospital, Stockholm, Sweden; 4 Department of Medicine, Haukeland University Hospital, Bergen, Norway; 5 Department of Intensive Care, Rigshospitalet, University of Copenhagen, Copenhagen, Denmark; 6 Laboratory of Molecular Medicine, Department of Clinical Immunology, Rigshospitalet, University of Copenhagen, Copenhagen, Denmark; 7 Department of Physiology and Pharmacology, Section for Anaesthesiology, Karolinska University Hospital, Stockholm, Sweden; 8 Department of Anaesthesiology and Intensive Care Medicine, Sahlgrenska University Hospital, Gothenburg, Sweden; 9 Department of Anaesthesia and Intensive Care, Blekingesjukhuset, Karlskrona, Sweden; 10 Helmholtz-Zentrum für Infektionsforschung, Braunschweig, Germany; 11 Department of Anaesthesiology, Haraldsplass Deaconess Hospital, Bergen, Norway

## Abstract

Early assessment of necrotising soft tissue infection (NSTI) is challenging. Analysis of inflammatory markers could provide important information about disease severity and guide decision making. For this purpose, we investigated the association between cytokine levels and the Laboratory Risk Indicator for Necrotising Fasciitis (LRINEC)-score, disease severity and mortality in NSTI patients. In 159 patients, plasma was analysed for IL-1β, IL-6, IL-10 and TNF-α upon admission. The severity of NSTI was assessed by SAPS, SOFA score, septic shock, microbial aetiology, renal replacement therapy and amputation. We found no significant difference in cytokine levels according to a LRINEC- score above or below 6 (IL-1β: 3.0 vs. 1.3; IL-6: 607 vs. 289; IL-10: 38.4 vs. 38.8; TNF-α: 15.1 vs. 7.8 pg/mL, *P* > 0.05). Patients with β-haemolytic streptococcal infection had higher level of particularly IL-6. There was no difference in mortality between patients with a LRINEC-score above or below 6. In the adjusted analysis assessing 30-day mortality, the association was strongest for IL-1β (OR 3.86 [95% CI, 1.43-10.40], *P* = 0.008) and IL-10 (4.80 [1.67-13.78], *P* = 0.004). In conclusion, we found no significant association between the LRINEC-score and cytokine levels on admission. IL-6 was consistently associated with disease severity, whereas IL-1β had the strongest association with 30-day mortality.

Necrotising soft tissue infection (NSTI) is a serious disease that causes necrotic lesions in any layer within the soft tissue compartments. The incidence of NSTI has increased over the past decades and the mortality and amputation rate remain high despite increased focus on these patients^1^,^2^. However, limited data exist on the disease burden in Scandinavia. A 13-year-old active surveillance, performed in Denmark, showed that invasive streptococcal infections had an incidence of 2.6 cases per 100,000 person-years and that NSTI accounted for 6%^3^. A retrospective study from western Norway reported an incidence of streptococcal NSTI of 1.4 cases per 100,000 person-years^4^. Even though NSTI is uncommon, many physicians will encounter at least one case during their working life^5^.

The pathogenesis of NSTI has not yet been fully elucidated, but toxin-induced inflammatory responses are believed to be involved in both tissue pathology and systemic toxicity^6^,^7^. Interleukin-1β (IL-1β), IL-6, IL-10 and tumor necrosis factor-α (TNF-α) are some of the classical sepsis-associated cytokines. We know that plasma levels of the pro-inflammatory cytokines (e.g. IL-1β, IL-6, TNF-α) and anti-inflammatory cytokines (e.g. IL-10) are elevated in patients with sepsis not surviving on Day 28 compared with the levels in survivors^8^. There are only few prospective studies aimed at characterizing the systemic cytokine responses in patients with NSTI and they are based on small samples, investigating fewer than 20 patients^9^,^10^. Further insight into the cytokine response profile in NSTI of varying severity and aetiology is needed to forward our understanding of the pathophysiology and to identify potential targets for improved diagnostics and interventions.

A key challenge is the presentation of the disease as it is often indistinguishable from non-necrotising infections^11^. Accordingly, the Laboratory Risk Indicator for Necrotising Fasciitis (LRINEC) score was developed as a diagnostic scoring system to detect early cases of NSTI^12^; a cut-off of a LRINEC score ≥6 was observed to have the highest predictive values^12^. The score has also been associated with clinical outcomes, including amputation and death^13^,^14^. However, other studies have been unable to find clear associations between the LRINEC score and clinically important outcomes^15^,^16^,^17^. Identifying biomarkers that reflect the inflammatory state in patients with NSTI would be desirable because such biomarkers might prove usefulness for prognostication and guide treatment. C-reactive protein (CRP) is one of the 6 variables included in the LRINEC score and has been found elevated in non-surviving patients with NSTI^14^. IL-6 induces CRP production during acute inflammation and might therefore be a relevant biomarker to elaborate on in this patient group.

Accordingly, we aimed to investigate the association between plasma levels of inflammatory cytokines and disease severity in patients with NSTI. We focused primarily on the association between baseline IL-6 level and the LRINEC score. We hypothesized that high IL-6 level upon hospital admission was associated with a high LRINEC score. Secondarily, we focused on the association between IL-1β, IL-6, IL-10 and TNF-α level and the simplified acute physiology score II (SAPS II) and Sepsis-related organ failure assessment (SOFA) score, septic shock, β-haemolytic streptococcal infection, renal replacement therapy (RRT), amputation and 30-day mortality.

## Results

**Figure 1:**
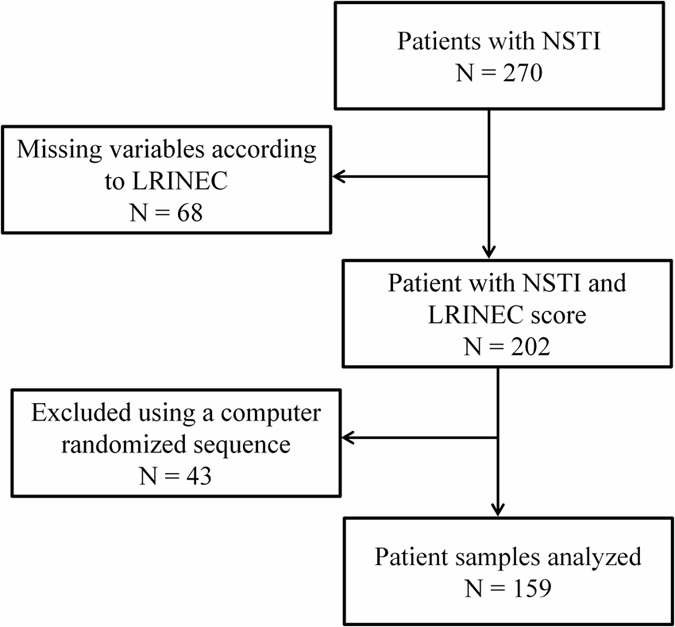
Flow chart of patient inclusion. Only patients with a LRINEC score had plasma analysed according to the primary analysis. LRINEC, Laboratory Risk Indicator for Necrotising Fasciitis; NSTI, Necrotising soft tissue infection.

We enrolled 270 patients with NSTI during the 33 months of inclusion between February 2013 and November 2015 of whom 68 had missing data for the LRINEC score (Fig. 1). Of the remaining 202 patients with a LRINEC score, we randomly selected 159 patients according to the sample size calculation and measured the plasma cytokine levels. One foreign patient was lost to follow-up as he left the country on Day 7 and was excluded from the survival analyses. In 31 patients, SAPS II could not be calculated due to missing values.

Table 1 shows baseline characteristics of the 159 included patients, which appeared to be similar between the groups with the exception of the variables included in the LRINEC score (CRP, sodium, creatinine) and organ diseases affecting these variables (chronic kidney disease). In 61% of the cases, the baseline samples of the cytokines were obtained after the first operation with a median of 3.5 hours. The rest of the samples were obtained just prior to surgery. There was no difference in baseline cytokine level in samples obtained prior to and after operation according to IL-1β (3.1 (IQR, 0, 7–9.1) vs. 2.1 (0.6–7.1) pg/mL, *P* = 0.54), IL-6 (733 (IQR, 116–3,090) vs. 381 (172–2,395) pg/mL, *P* = 0.64), IL-10 (50.3 (IQR, 11.1–198.3) vs. 36.5 (9.9–80.2) pg/mL, *P* = 0.13) and TNF-α (15.2 (IQR, 5.0–59.7) vs. 13.1 (3.6–37.4) pg/mL, *P* = 0.32).

### LRINEC score

**Figure 2:**
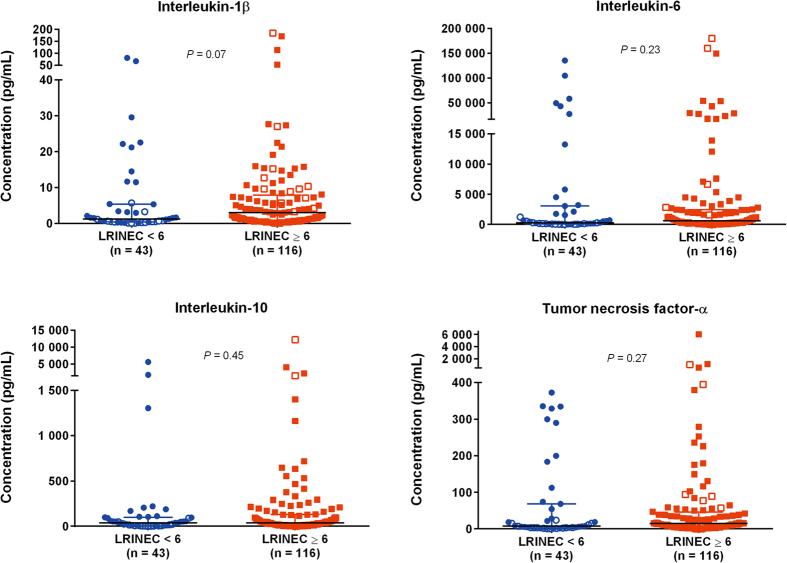
Levels of plasma cytokines on admission according to the LRINEC score in patients with necrotising soft tissue infection. LRINEC, Laboratory Risk Indicator for Necrotising Fasciitis. Filled circles and squares indicate patients with septic shock. Median with interquartile range is illustrated. Comparisons were performed using the Mann-Whitney *U* test.

A LRINEC score ≥6 was seen in 116 (73%) patients. Those with a LRINEC score ≥6 had a mean IL-6 level of 8,006 pg/mL, whereas those with a score <6 had a mean IL-6 level of 10,729 pg/mL (mean difference 2,723 pg/mL, 95% CI [−6,947 to 12,393], *P* = 0.58). Likewise, no significant difference was found in any of the cytokine levels between the two groups using a Mann-Whitney *U*-test and median values (Fig. 2). In line with this, we found no significant correlation between the baseline levels of the cytokines and the LRINEC score (Table 2). In contrast, all cytokines showed a significant correlation with the severity scores of SAPS II and SOFA, as well as with CRP, creatinine and lactate with IL−6 demonstrating the highest correlation with these variables. The cytokine levels were significantly greater in the infected patients than in the control patients (Table 3). Moreover, patients with NSTI and septic shock at baseline had significantly higher levels of IL-1β, IL-6, IL-10 and TNF-α level than in patients with NSTI without shock. Patients with a LRINEC score ≥6 did not have a significantly higher presence of septic shock compared with patients with a LRINEC score <6 nor did they have a higher rate of death at Day 30 (Table 1).

### Microbiology

**Figure 3:**
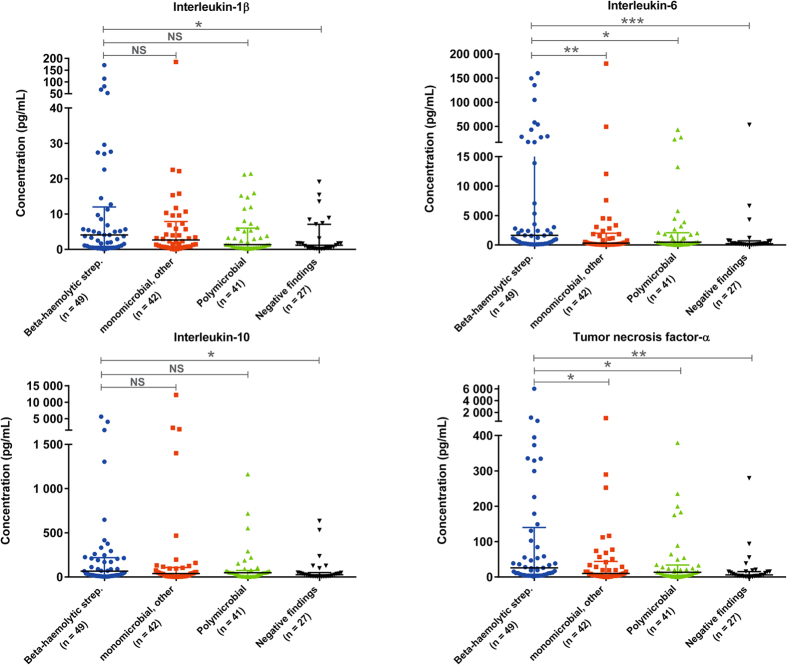
Levels of plasma cytokines on admission according to microbial aetiology in patients with necrotising soft tissue infection. Median with interquartile range is illustrated. Comparisons were performed using the Mann-Whitney *U* test. NS, non-significant; *p < 0.05; **p < 0.01; ***p < 0.0001. Monomicrobial infection, other includes *Staphylococcus aureus, Enterococcus faecium, Escherichia coli, Clostridium septicum, Clostridium perfringens, Corynebacterium amycolatum* or gram-negative rods (unspecified). In 19 of the 41 polymicrobial cases, β-haemolytic *Streptococcus* was part of the infections (group A, n = 11; group B, n = 3; group C, n = 2; group G, n = 2; group C + G, n = 1).

Infection with β-haemolytic *Streptococcus* was the most common monomicrobial finding in the patients (n = 49, Table 1). Of these, group A was present in 39 patients, group B in one patient, group C in 4 patients and group G in 5 patients. The cytokine levels according to microbiology are illustrated in Fig. 3. Compared with patients with other bacterial infections, those with β-haemolytic streptococcal infection had higher levels of IL-6, IL-10 and TNF-α but not a higher IL-1β level (Table 3). There was no significant difference between patients infected with β-haemolytic *Streptococcu*s or non-*Streptococcus* according to amputation (14% [95% CI, 7–27] vs. 20% [95% CI, 14–29], *P* = 0.39) and 30-day mortality (12% [95% CI, 5–25] vs. 17% [95% CI, 11–26], *P* = 0.41). However, patients infected with β-haemolytic *Streptococcu*s were more likely to have septic shock than were non-*Streptococcus* infected patients (84% [95% CI, 71–92] vs. 68% [95% CI, 59–76], *P* = 0.04). Moreover, patients with the presence of bacteraemia, defined as having a positive blood culture, had higher levels of IL-6 (1296 vs. 380 pg/mL, *P* = 0.01) and TNF-α (24.78 vs. 11.97 pg/mL, *P* = 0.02) than those with negative blood cultures. No difference was found according to IL-1β (2.47 vs. 1.88 pg/mL, *P* = 0.25) or IL-10 (44.31 vs. 37.99 pg/mL, *P* = 0.49).

### Clinical outcomes

The 30-day mortality was higher if baseline cytokine levels were above the median (Table 4). The odds ratio was larger for IL-10, but IL-1β proved to be more consistent in predicting 30-day mortality when adjustments for baseline risk factors were performed. The diagnostic value for the prediction of 30-day mortality was the highest for IL-1β and IL-10 (Table 5). Patients who subsequently received RRT had higher median levels of IL-1β, IL-6, IL-10 and TNF-α (Table 3) than did those not receiving RRT. Patients who underwent amputation did not have a higher median IL-1β level but had higher median levels of IL-6, IL-10 and TNF-α compared with those not amputated (Table 3).

### The cytokine response to surgery

**Figure 4:**
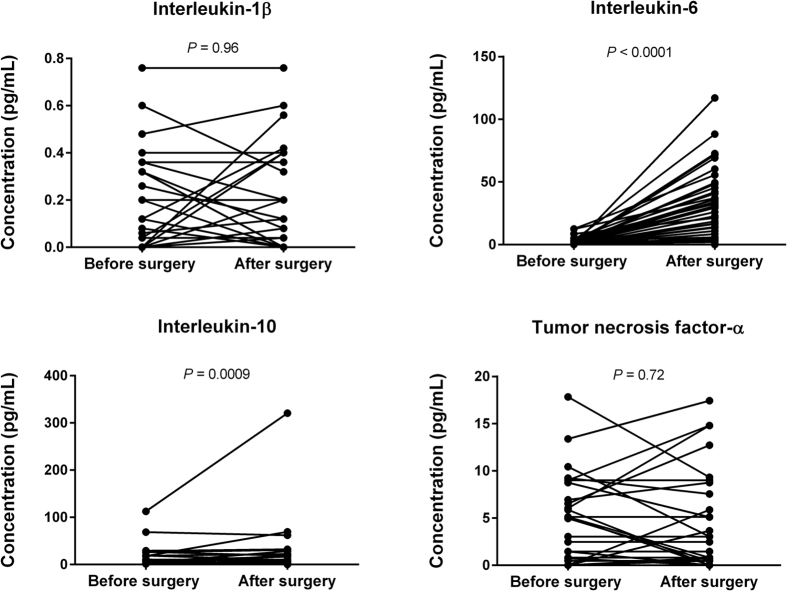
Levels of plasma cytokines in 43 non-infected control patients before surgery (baseline) and after surgery (2–6 h postoperatively). Comparisons were performed using the Wilcoxon signed-rank test.

The 43 non-infected control patients (61% men) had a median age of 63 years (IQR, 45–71), CRP of 3 mg/L (IQR, 1–5) and a leucocyte count of 7.1 10^9^/L (IQR, 6.3–9.8). They experienced a significant increase in IL-6 and IL-10 levels after surgery compared with samples taken before surgery, whereas the levels of IL-1β and TNF-α were not significantly altered by surgery (Fig. 4).

## Discussion

In this prospective study of patients with NSTI, we found that baseline cytokine levels were not significantly different in patients with a LRINEC score ≥6 compared with a score <6. Notably, the cytokine levels were associated with the severity of infections as evident by significantly elevated levels in patients with septic shock and positive correlations with the severity scores (SAPS II and SOFA). Moreover, there was an association between high cytokine levels and subsequent use of RRT, amputation and 30-day mortality.

NSTI is a difficult diagnosis for the clinicians to make. This is evident in our prospective study of NSTI patients with only 15% having crepitus, 25% having gas visualized with CT and 33% having severe pain and requiring opioid (Table 1). Even though the LRINEC score was designed to discriminate between NSTI and non-NSTI, it is primarily believed to be useful in the context of strongly suspected NSTI^5^,^18^. Our data suggest that the score should be used with caution as only 73% of patients with NSTI had a LRINEC score of ≥6. However, this needs to be investigated further in a larger patient cohort. Additionally, among the baseline data presented in Table 1, variables included in the LRINEC score, i.e. sodium, creatinine and CRP, were significantly higher in patients with a score of ≥6 as a consequence of the stratification but this was not the case with the severity scores or infection outcome measures. We did not detect a significant difference in baseline IL-6 level in patients with a score <6 vs. ≥6, but the rather wide 95% CI indicates a risk of overlooking a clinically relevant difference in IL-6 level as defined in our sample size calculation. This is related to the large variability in IL-6 level and in line with other studies of severe infections^19^,^20^.

CRP is included in the LRINEC score and often used to monitor disease progression in patients with NSTI. CRP is released from hepatic cells after stimulation of IL-6 amongst others^21^. Because we found a significant difference in CRP levels between the groups of high vs. low LRINEC score, a similar trend for LRINEC and IL-6 would be expected. The lack of an association could be related to the time course of the inflammatory state as IL-6 levels peak within hours of infection, whereas CRP reaches its peak after around 48 hours^22^. However, this is unlikely as IL-6 was significantly higher in patients with septic shock, β-haemolytic streptococcal infection and in patients undergoing amputation or given RRT. Moreover, the median time from diagnosis of NSTI until blood sampling was 3.5 hours with no significant difference between patients with high and low LRINEC scores (Table 1). It seems more plausible that the LRINEC score does not reflect the inflammatory state in the patients. This is an important finding because it might explain why several studies have failed to prove an association between a high LRINEC score and the presence of NSTI and disease severity^15^,^16^,^17^,^23^,^24^. This study was not designed to evaluate the diagnostic value of the LRINEC score. Instead we focused on the potential value of the score as a measure of disease severity and the ability to predict adverse outcomes of NSTI. The data are important as they dispute earlier claims of the LRINEC score being able to predict disease severity^13^,^14^. A natural next step would be to conduct a study with the inclusion of patients with cellulitis or erysipelas to test whether the cytokine profiles are able to distinguish between NSTI and these diseases. In addition, future studies should assess the usefulness of admission IL-6 and IL-1β determination as prognostic markers used alone or in combination with the LRINEC score. This was not possible with the current study as blood samples used in the LRINEC score were obtained at a different time point than the samples used for cytokine analyses.

NSTI is classified based on the microbiological aetiology in type 1 (polymicrobial, 70–80%) and type 2 (monomicrobial, 20–30%)^7^. In contrast to previous studies and the general belief that NSTI is predominantly polymicrobial^5^,^25^, more patients in our cohort had a monomicrobial infection. We found β-haemolytic *Streptococcus*, predominantly group A, to be the most common causative microorganism, which is in line with two large retrospective studies of 198 and 89 patients with NSTI^26^,^27^. We found significantly higher IL-6 and TNF-α levels in streptococcal infected patients than in patients infected with other agents. This is consistent with septic shock being more prevalent in streptococcal infected patients and levels of these two cytokines being elevated in our septic shock cohort. This suggests that plasma levels of IL-6 and TNF-α might be of value clinically to identify NSTI cases with systemic inflammation and potentially also as an indication for immunomodulatory treatments such as intravenous polyspecific immunoglobulin (IVIG). Although the efficacy of IVIG therapy in sepsis and septic shock has been questioned^28^, certain subgroups of patients with sepsis, such as group A streptococcal toxic shock syndrome, have been reported to benefit from the treatment^29^,^30^,^31^. However, it should be noted that in regards to IVIG treatment in NSTI, the clinical evidence is limited to a few case reports and case series^32^,^33^. Taken together, the IL-6 and TNF-α data seem particularly interesting in light of group A streptococcal NSTI, which is often complicated by septic shock and associated with substantial mortality. However, more research is needed on these topics.

Our study of NSTI reveals that the infection is characterized by a strong systemic inflammatory response. Considering that surgery is a traumatic insult that elicits an inflammatory response, we chose to include a control group of non-infected surgical patients. All control samples had significantly lower levels of cytokines compared with the NSTI patients. Notably, comparison of pre- and post-surgery samples revealed a significant but limited increase in IL-6 and IL-10 levels after surgery, whereas no such difference was seen for IL-1β and TNF-α levels. This illustrates how complex and specific the cytokine response profile is in response to different trauma or stimuli. In the non-infected surgical control persons, IL-6 increased from 0 to 24 pg/mL after surgery compared with the median of 539 pg/mL in the NSTI patients. Thus, the surgery itself is less likely to contribute significantly to the increase in IL-6 and IL-10 as seen in the infected patients.

A notable limitation to this study is the measurements of only baseline cytokine values. It would have been interesting to investigate the fluctuations in the concentrations over time to elucidate the changes in the biological processes. However, the baseline concentrations of the cytokines are the most important values for the clinicians as these often guide the course of treatment in the acute phase of the disease. Another limitation is that only four different cytokines were analysed. However, we chose well-recognized markers associated with severity of sepsis as some of the inflammatory processes probably reflect those in patients with NSTI. We could have included several other markers, but this would have increased the risk of chance findings. In addition, it is often difficult to correlate the findings of significant biomarkers with the bed-side use. Only few markers have been investigated in patients with NSTI and even fewer are used in clinical practice. Interestingly, IL-1β proved to be an independent predictor for 30-day mortality. Further support for IL-1β as a potentially useful marker for NSTI was recently provided in a murine model of streptococcal NSTI, in which IL-1β response network was identified as a key network involved in modulating severity of streptococcal NSTI^34^. Another limitation to this study is the limited time of follow-up regarding amputation. We only have data from admission and the following 7 days (defined a priori). We might have overlooked relevant amputations performed at later stage. We chose 7 days as follow-up because we only wanted to include amputation related specifically to the infection. Importantly, it is our experience from the daily clinical settings in all centres that only few of the patients are amputated after the first week of infection. Thus, our data provide a realistic picture of the disease course.

We believe that a major future challenge is to identify relevant biomarkers and combine them with clinical scores to detect patients with NSTI and improve risk stratification. In this study, we found that IL-1β, IL-6, IL-10 and TNF-α might be of value in the risk stratification of patients with NSTI with the purpose of enhancing prognostication and decision making in these critically ill patients. Furthermore, we found variations in the cytokine responses in NSTIs depending on microbial aetiology, suggesting that different pathogenic mechanisms contribute to the disease. Future studies should assess the usefulness of admission IL-6 and IL-1β determination as prognostic and diagnostic markers in combination with the LRINEC score.

In conclusion, we find that the LRINEC score is not significantly associated with the cytokine response in patients with NSTI. In contrast, baseline plasma cytokine levels correlate well with 30-day mortality and disease severity assessed by SAPS II and SOFA score, the presence of septic shock, infection with β-haemolytic *Streptococci* and subsequent use of RRT and amputation. In the adjusted analyses, IL-1β and IL-10 levels proved to have the strongest association with 30-day mortality.

## Materials and Methods

### Study design

This multicentre, prospective, observational study was approved by the ethics committees and Data-Protection agencies in Denmark (the National Committee on Health Research Ethics: 1211709; the regional ethics committee: H-2-2014-071), Sweden (the regional ethics committee in Gothenburg; 930-12) and Norway (the regional committee for medical and health research ethics, Bergen; 2012/2227). We included patients admitted to five Scandinavian hospitals: Righospitalet (Copenhagen, Denmark), Karolinska University Hospital (Stockholm, Sweden), Blekingesjukhuset (Karlskrona, Sweden), Sahlgrenska University Hospital (Gothenburg, Sweden) and Haukeland University Hospital (Bergen, Norway). Written informed consent was obtained from all patients or their next of kin as soon as possible. The study is part of the EU-funded INFECT project and is registered at ClinicalTrials.gov (NCT01790698). Data from some patients in the Danish cohort have been reported elsewhere^35^,^36^. The study was carried out in accordance with the relevant guidelines and regulations.

### Study patients

#### Patients with NSTI

We included patients 18 years of age or older with suspected necrosis engaging any layer of the soft tissue compartments who had either been admitted to the ICU or undergone surgery for NSTI. We excluded patients in whom surgery revealed no necrosis.

#### Non-infected surgical control patients

In order to elucidate the cytokine response to surgery and evaluate our sampling method, we included patients 18 years of age or older who underwent elective surgery at Rigshospitalet (University Hospital of Copenhagen, Denmark). Patients with ongoing infection or inflammatory conditions were excluded (assessed by diagnoses in the medical records and by CRP levels on hospital admission). Forty-three control patients, matched for age and sex, were included between September 2014 and March 2015.

### Data collection

The following data were obtained from patient medical records by the study trial investigators or their co-workers: baseline patient characteristics, physiology values, biochemistry, microbiology, chronic diseases, disease severity scores (LRINEC, SAPS II, SOFA), the presence of septic shock (defined as use of vasopressors), use of RRT in the intensive care unit (ICU), and whether the patient had undergone amputation during the first seven days of admission. Vital status data were obtained from national registries. All data were entered into a centralized clinical database. LRINEC was calculated from 6 variables (C-reactive protein, total white cell count, haemoglobin, sodium, creatinine, glucose), a score ≥6 indicating a high risk of NSTI, amputation rate and mortality^12^,^13^,^37^. SAPS II was calculated from 17 variables, with a higher score indicating more severe disease^38^. SOFA score was calculated based on sub-scores for each of five components: respiratory system, circulatory system, liver, kidneys and coagulation, with higher scores indicating more severe organ failure^39^.

Patients had blood sampled from an arterial line into EDTA tubes upon admission (baseline). All values included in the LRINEC score were obtained prior to the first operation. Non-infected control patients had venous blood sampled before surgery (baseline) and after surgery (2–6 h postoperatively). The latter sample was taken to assess the effect of the surgical trauma on the inflammatory response. All samples were immediately put on ice and centrifuged within 40 minutes. The collected plasma was stored at −80 °C until processing.

### Multiplex bead array assays

Analyses were performed in February 2016, thus three months after enrolment of the final patient with NSTI and 11 months after the final control patient. Plasma levels of IL-1β, IL-6, IL-10 and TNF-α were determined using multiplex bead assays (Bio-Rad Laboratories, Hercules, CA, USA; Bio-Plex Pro^TM^ assay) according to the manufacturer’s instructions. Analyses were done using the Bio-Plex^®^ MAGPIX^TM^ Multiplex Reader (Bio-Rad Laboratories, Hercules, CA, USA). The samples were diluted in 1:4 and 1:10 against a standard pool with known concentration and the mean values were calculated after correction for the diluting steps. According to the manufacturer, the detection range without dilution was: IL-1β (0.2–556 pg/mL), IL-6 (5.2–18,618 pg/mL), IL-10 (1.1–11,850 pg/mL) and TNF-α (0.2–2,059 pg/mL) with an intra-assay variation of 8% and an inter-assay variation of 10%. Measurements below detection limit were set to 0. One patient had an IL-6 level above detection range, when taking the dilution factor into account, and was set to 180,000 pg/mL.

### Outcome measures

According to our published protocol^40^, the primary analysis was baseline IL-6 level in patients with NSTI and a LRINEC score <6 vs. ≥6. IL-6 was chosen because it is an important mediator of the acute inflammatory response and a recognized biomarker in sepsis^8^. A LRINEC cut-off ≥6 was chosen because it has proven to have the highest predictive values and because of an expected higher amputation and mortality rate, making it clinically relevant^12^,^13^. Secondary analyses included the association between baseline IL-1β, IL-6, IL-10 and TNF-α level and SAPS II, SOFA score, septic shock on Day 1 in the ICU, β-haemolytic streptococcal infection, use of RRT in the ICU, amputation within the first seven days of admission and 30-day mortality. There was no standardized protocol for use of RRT or amputation.

### Statistical analysis

Categorical data are presented as absolute numbers (%) and continuous data as medians (IQR). For the primary analysis, the unpaired Student *t*-test was applied and quantified by a 95% confidence interval (CI). Comparisons were performed using unadjusted χ^2^ tests for binary outcome measures, Mann-Whitney *U* tests for unpaired analyses and Wilcoxon-rank tests were used for paired analyses. We assessed correlations by Spearman’s rank correlation tests. We used logistic regression analyses with odds ratios (OR) and 95% CI to assess the correlation between baseline biomarker levels and 30-day mortality and adjusted for differences in baseline variables (sex, age, SAPS II and chronic disease [diabetes, liver cirrhosis, chronic kidney disease, cardiovascular disease, chronic obstructive pulmonary disease, peripheral vascular disease, immune deficiency, malignancy, rheumatoid disease]). Receiver operating characteristic (ROC) curves were analysed for 30-day mortality. Patients with missing SAPS II data were excluded from the part of the multiple logistic regression analysis where adjustment for SAPS II was performed.

Analyses were performed with Statistical Package for the Social Sciences 22.0 software (SPSS Inc., Chicago, IL, USA) and GraphPad Prism 6.0 software (GraphPad Inc., La Jolla, CA, USA). P-values <0.05 were considered to indicate statistical significance.

### Sample size

We considered a difference in IL-6 level of 1000 pg/mL as clinically relevant because previous studies have found similar differences between sepsis patients according to shock^19^,^20^. In a pilot study of seven patients with NSTI, we found a standard deviation in IL-6 on admission of 2000 pg/mL. Based on this, we decided to include 159 patients as this would allow the detection of a difference of 1000 pg/mL with a statistical power of at least 85% at the 5% significance level. The 159 patients were selected randomly out of the total cohort of 202 NSTI patients with a LRINEC score using a computer-generated allocation table.

## Additional Information

**How to cite this article**: Hansen, M. B. *et al*. Association between cytokine response, the LRINEC score and outcome in patients with necrotising soft tissue infection: a multicentre, prospective study. *Sci. Rep.* **7**, 42179; doi: 10.1038/srep42179 (2017).

**Publisher's note:** Springer Nature remains neutral with regard to jurisdictional claims in published maps and institutional affiliations.

## Figures and Tables

**Table 1:** Baseline characteristics of patients with necrotising soft tissue infection for the entire study cohort and according to the LRINEC score.

	Study cohort (*n* = 159)	LRINEC <6 (*n* = 43)	LRINEC ≥6 (*n* = 116)	*P*-value
Age, years	61 (53–69)	60 (47–68)	61 (54–69)	0.30
Sex, male	95 (60)	27 (63)	68 (59)	0.63
**Chronic disease**				
Diabetes	34 (21)	5 (12)	29 (25)	0.07
Liver cirrhosis	7 (4)	2 (5)	5 (4)	0.93
Chronic kidney disease	16 (10)	0 (0)	16 (14)	0.01
Cardiovascular disease	68 (43)	16 (37)	52 (45)	0.39
COPD	16 (10)	6 (14)	10 (9)	0.38
Peripheral vascular disease	21 (13)	1 (2)	20 (17)	0.01
Immune deficiency (AIDS)	1 (1)	1 (2)	0 (0)	0.30
Malignancy	23 (15)	4 (9)	19 (16)	0.26
Rheumatoid disease	11 (7)	3 (7)	8 (7)	0.99
Steroid treatment	19 (12)	8 (19)	11 (10)	0.12
**Symptoms**				
Severe pain requiring opioid	52 (33)	13 (30)	39 (34)	0.92
Palpable gas (crepitus)	24 (15)	4 (9)	20 (17)	0.45
Gas visualized with CT	40 (25)	9 (21)	31 (27)	0.38
**Cytokines, pg/mL**				
IL-1β	2.3 (0.7–7.3)	1.3 (0.4–5.4)	3.0 (0.8–7.9)	0.07
IL-6	539 (156–2,484)	289 (88–3,050)	607 (181–2,473)	0.23
IL-10	38.8 (10.0–115.4)	38.8 (6.7–97.9)	38.4 (11.7–129.7)	0.45
TNF-α	15.1 (4.3–49.8)	7.8 (3.1–68.2)	15.1 (5.3–45.8)	0.26
Time from diagnosis to baseline blood sample (hours)	3.5 (1.5–8.75)	3.25 (1.0–11.75)	3.75 (1.75–8.75)	0.49
**Body part affected on admission**				
Head/neck	12 (7)	4 (9)	8 (7)	0.54
Thorax	6 (4)	0 (0)	6 (5)	0.19
Abdomen	3 (2)	1 (2)	2 (2)	0.21
Extremities	65 (41)	22 (51)	43 (37)	0.11
Genital/perineum	33 (21)	9 (21)	24 (21)	0.28
Multiple body regions	40 (25)	7 (17)	33 (28)	0.12
**Microorganism in blood and/or tissue**				
β-haemolytic *Streptococcus*	49 (31)	12 (28)	37 (32)	0.63
*Staphylococcus aureus*	6 (4)	2 (5)	4 (3)	0.66
Gram-negative bacteria	13 (8)	4 (9)	9 (8)	0.75
Monomicrobial, other	23 (14)	7 (16)	16 (14)	0.69
Polymicrobial	41 (26)	10 (23)	31 (27)	0.66
Negative findings	27 (17)	8 (19)	19 (16)	0.74
**Biochemistry**				
Leukocyte count (highest), 10^9^/L	15.9 (9.4–23.8)	15.9 (7.6–21.5)	15.9 (9.7–24.3)	0.71
C-reactive protein (highest), mg/L	201 (126–296)	96 (54–197)	226 (159–306)	<0.0001
Potassium (highest), mmol/L	4.3 (3.9–4.8)	4.2 (4.0–4.7)	4.4 (3.9–4.8)	0.83
Sodium (lowest), mmol/L	135 (131–138)	137 (134–139)	134 (131–137)	0.005
Creatinine (highest), μmol/L	121 (77–209)	91 (64–129)	145 (80–233)	0.001
pH (lowest)	7.31 (7.22–7.37)	7.36 (7.23–7.40)	7.30 (7.2–7.4)	0.05
Lactate (highest), mmol/L	2.3 (1.3–4.2)	1.9 (1.1–4.1)	2.4 (1.4–4.4)	0.26
**Outcomes and ICU scores**				
SAPS II^a^	46 (35–55)	47 (31–58)	45 (36–52)	0.92
SOFA score^b^ (Day 1)	8 (5–10)	6 (3–10)	8 (6–10)	0.17
Septic shock^c^ (Day 1)	116 (73)	28 (65)	88 (76)	0.18
Ventilation within 7 d	142 (89)	34 (79)	108 (93)	0.019
RRT within 7 d	40 (25)	12 (28)	28 (24)	0.63
Amputation of extremity within 7 d	29 (18)	8 (19)	21 (18)	0.94
30-day mortality, % (95% CI)	25 (16, 11–22)	10 (23)	15 (13)	0.24
90-day mortality, % (95% CI)	34 (21, 16–28)	12 (28)	22 (19)	0.41

COPD, chronic obstructive pulmonary disease; CT, computed tomography; SAPS II, simplified acute physiology score II; SOFA, sequential organ failure assessment; LRINEC, laboratory risk indicator for necrotising fasciitis; ICU, intensive care unit; RRT, renal replacement therapy. Values denote median (25–75 percentiles) or number (%). Comparisons between LRINEC <6 and ≥6 were performed with Mann-Whitney *U* or X^2^-test. ^a^Patients with missing value: n = 31. ^b^Patients with missing value: n = 14; the SOFA score was modified as the Glasgow Coma Scale was not assessed. ^c^Septic shock defined as the use of any inotropica or vasopressor.

**Table 2:** Spearman rank correlation between variables and baseline biomarker levels in patients with necrotising soft tissue infection.

	IL-1β		IL-6		IL-10		TNF-α	
	*Rho*	*P*	*Rho*	*P*	*Rho*	*P*	*Rho*	*P*
LRINEC	0.12	0.14	0.10	0.24	0.09	0.29	0.05	0.55
SAPS II	0.30	0.001	0.41	<0.0001	0.34	<0.0001	0.33	<0.0001
SOFA score	0.34	<0.0001	0.51	<0.0001	0.42	<0.0001	0.44	<0.0001
Leucocyte count	−0.02	0.81	−0.08	0.31	−0.05	0.57	−0.13	0.11
CRP	0.18	0.03	0.17	0.05	0.04	0.67	0.09	0.29
Creatinine	0.34	<0.0001	0.47	<0.0001	0.37	<0.0001	0.38	<0.0001
Lactate	0.58	<0.0001	0.70	<0.0001	0.58	<0.0001	0.64	<0.0001

LRINEC, laboratory risk indicator for necrotising fasciitis; SAPS II, simplified acute physiology score II; SOFA, sequential organ failure assessment; CRP, c-reactive protein.

**Table 3:** Differences in median baseline cytokine levels and disease severity in patients with necrotising soft tissue infection.

	IL-1β	P	IL-6	P	IL-10	P	TNF-α	P
Septic shock	3.1 (1.0–8.3)	0.0008	982 (233–3475)	<0.0001	50.4 (17.8–183.7)	<0.0001	19.5 (6.0–55.5)	<0.0001
No septic shock	0.9 (0.4–5.7)		156 (69–541)		14.7 (4.8–42.2)		5.3 (3.1–15.2)	
β-haemolytic strep.	4.1 (0.9–12.0)	0.052	1628 (298–15611)	<0.0001	66.2 (16.1–218.5)	0.03	26.1 (7.6–140.1)	0.007
Other infections^a^	1.7 (0.6–6.9)		313 (117–1778)		36.5 (8.4–93.2)		11.5 (3.5–30.0)	
RRT^b^	4.9 (1.3–11.9)	0.02	2649 (284–12942)	<0.0001	71.6 (11.6–243.3)	0.03	48.4 (9.1–174.9)	<0.001
No RRT	1.7 (0.6–5.7)		373 (129–1618)		35.3 (9.4–87.8)		11.2 (3.7–28.0)	
Amputation^b^	3.0 (1.3–11.5)	0.12	2019 (364–5799)	0.02	79.2 (39.0–330.9)	0.0008	33.8 (5.2–107.6)	0.04
No amputation	1.8 (0.6–7.2)		420 (140–1952)		33.7 (8.8–95.6)		12.5 (4.3–35.1)	
NSTI	2.3 (0.7–7.3)	<0.0001	539 (156–2484)	<0.0001	38.8 (10.0–115.4)	<0.0001	15.2 (4.3–49.8)	<0.0001
Surgical controls	0.0 (0.0–0.7)		0.0 (0.0–2.4)		1.2 (0.2–8.3)		0.8 (0.0–17.8)	

Strep., *streptococcus* infection; RRT, renal replacement therapy; NSTI, necrotising soft tissue infection. ^a^All other types of microorganisms than monomicrobial β-haemolytic strep. infection. ^b^Within the first 7 days of admission.

**Table 4:** Univariate and multivariate logistic regression analyses of 30-day mortality in patients with necrotising soft tissue infection based on high vs. low concentrations of the cytokines according to median values.

	Unadjusted*			Adjusted for age, sex, chronic disease*			Adjusted for age, sex, chronic disease, SAPS II**		
	OR	95% CI	*P*	OR	95% CI	*P*	OR	95% CI	*P*
**IL-1β**									
Low ≤ 2.3 ng/mL	1			1			1		
High > 2.3 ng/mL	3.97	1.49–10.58	0.006	3.86	1.43–10.40	0.008	3.84	1.03–14.31	0.045
**IL-6**									
Low ≤ 539.2 ng/mL	1			1			1		
High > 539.2 ng/mL	2.52	1.01–6.21	0.047	2.70	1.07–6.83	0.036	1.56	0.48–5.11	0.46
**IL-10**									
Low ≤ 38.8 μg/mL	1			1			1		
High > 38.8 μg/mL	5.17	1.83–14.61	0.002	4.80	1.67–13.78	0.004	2.79	0.74–10.63	0.13
**TNF-α**									
Low ≤ 15.1 μg/mL	1			1			1		
High > 15.1 μg/mL	2.75	1.11–6.81	0.029	2.64	1.04–6.69	0.041	1.55	0.46–5.20	0.48

IL, interleukin; OR, odds ratio; CI, confidence interval; SAPS II, simplified acute physiology score II. ^*^One patient was excluded because of loss to follow-up due to emigration. ^**^31 patients were not included in the analysis due to missing data of SAPS II (4 of these died within the first 30 days). When the missing variables were replaced with the minimal value and maximum value, the median SAPS II was 38 (27–47) and 55 (41–70), respectively.

**Table 5:** Diagnostic accuracy of high baseline biomarker levels in predicting 30-day mortality in patients with necrotising soft tissue infection.

	IL-1β	IL-6	IL-10	TNF-α	LRINEC	SAPS II
Sensitivity	0.76 (0.56–0.90)	0.68 (0.48–0.84)	0.80 (0.60–92)	0.68 (0.48–0.84)	0.60 (0.41–0.77)	0.84 (0.65–0.95)
Specificity	0.56 (0.52–0.58)	0.54 (0.50–0.57)	0.56 (0.53–0.59)	0.56 (0.53–0.59)	0.25 (0.21–0.28)	0.59 (0.55–0.61)
PPV	0.24 (0.18–0.29)	0.22 (0.15–0.27)	0.26 (0.19–0.30)	0.23 (0.16–0.28)	0.13 (0.09–0.17)	0.30 (0.23–0.33)
NPV	0.93 (0.86–0.97)	0.90 (0.84–0.95)	0.94 (0.88–0.98)	0.90 (0.84–0.95)	0.77 (0.66–0.87)	0.95 (0.88–0.98)
LR for positive test	1.71 (1.17–2.14)	1.48 (0.97–1.95)	1.83 (1.28–2.23)	1.56 (1.01–2.06)	0.80 (0.52–1.07)	2.05 (1.44–2.44)
LR for negative test	0.43 (0.18–0.85)	0.59 (0.28–1.03)	0.36 (0.13–0.75)	0.57 (0.27–0.99	1.61 (0.81–2.78)	0.27 (0.09–0.64)
Area under ROC curve	0.70 (0.60–0.80)	0.70 (0.58–0.82)	0.70 (0.59–0.80)	0.68 (0.55–0.81)	0.43 (0.30–0.56)	0.80 (0.71–0.90)

PPV, positive predictive value; NPV, negative predictive value; LR, likelihood ratio; ROC, receiver operating characteristic; IL, interleukin; LRINEC, Laboratory Risk Indicator for Necrotizing Fasciitis; SAPS II, Simplified Acute Physiology Score. Data are presented as fractions (95% confidence interval). The prevalence of 30-day mortality was 16%. High baseline level defined as being above the median value.
